# *catena*-Poly[[diaqua­calcium(II)]-di-μ-2-chloro­nicotinato]

**DOI:** 10.1107/S1600536808044267

**Published:** 2009-01-10

**Authors:** Li Quan, Handong Yin, Liansheng Cui, Minglei Yang

**Affiliations:** a College of Chemistry and Chemical Engineering, Liaocheng University, Shandong 252059, People’s Republic of China

## Abstract

The itle compound, [Ca(C_6_H_3_ClNO_2_)_2_(H_2_O)_2_]_*n*_, contains polymeric chains extending along [100] that are generated by inversion centres. The Ca^2+^ ions are bridged by 2-chloronicotinate groups and exhibit an eight-coordination by six carboxylate O atoms of four different 2-chloronicotinate ligands and two O atoms of water molecules. In the crystal structure, inter­molecular O—H⋯O, O—H⋯N and C—H⋯O hydrogen bonds result in the formation of a supra­molecular network structure. The π–π contacts between the 2-chloro­nicotinate rings [centroid–centroid distances = 3.875 (3) and 3.701 (3) Å] may further stabilize the structure.

## Related literature

For general background, see: Schmidbaur *et al.* (1989 ▶, 1990 ▶). For related structures, see: Murugavel & Banerjee (2003 ▶); Radanovic *et al.* (2004 ▶). For bond-length data, see: Allen *et al.* (1987 ▶).
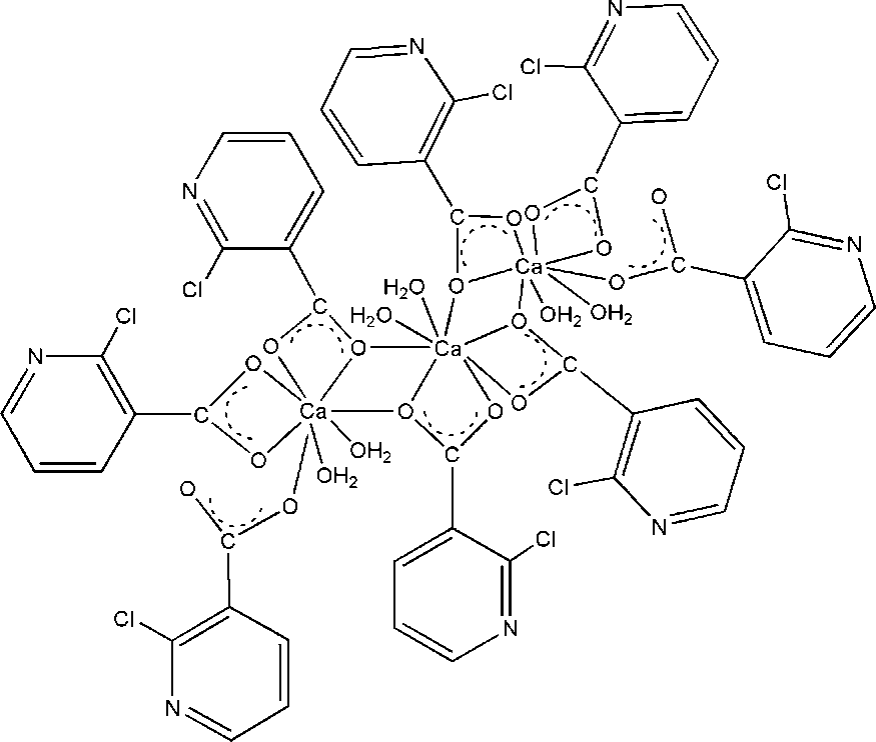


## Experimental

### 

#### Crystal data

[Ca(C_6_H_3_ClNO_2_)_2_(H_2_O)_2_]
*M* *_r_* = 389.20
Triclinic, 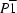

*a* = 7.8363 (10) Å
*b* = 10.8421 (16) Å
*c* = 10.8834 (16) Å
α = 98.455 (2)°
β = 97.610 (1)°
γ = 97.289 (1)°
*V* = 896.4 (2) Å^3^
*Z* = 2
Mo *K*α radiation
μ = 0.68 mm^−1^
*T* = 298 (2) K
0.48 × 0.40 × 0.30 mm

#### Data collection

Bruker SMART CCD area-detector diffractometer
Absorption correction: multi-scan (*SADABS*; Sheldrick, 1996 ▶) *T*_min_ = 0.738, *T*_max_ = 0.823
4594 measured reflections
3074 independent reflections
2306 reflections with *I* > 2σ(*I*)
*R*_int_ = 0.022

#### Refinement

*R*[*F* ^2^ > 2σ(*F* ^2^)] = 0.043
*wR*(*F* ^2^) = 0.145
*S* = 1.03
3074 reflections
208 parameters
H-atom parameters constrained
Δρ_max_ = 0.47 e Å^−3^
Δρ_min_ = −0.45 e Å^−3^

### 

Data collection: *SMART* (Siemens, 1996 ▶); cell refinement: *SAINT* (Siemens, 1996 ▶); data reduction: *SAINT*; program(s) used to solve structure: *SHELXS97* (Sheldrick, 2008 ▶); program(s) used to refine structure: *SHELXL97* (Sheldrick, 2008 ▶); molecular graphics: *ORTEP-3* (Farrugia, 1997 ▶) and *DIAMOND* (Brandenburg, 1998 ▶); software used to prepare material for publication: *SHELXTL* (Sheldrick, 2008 ▶).

## Supplementary Material

Crystal structure: contains datablocks I, global. DOI: 10.1107/S1600536808044267/hk2578sup1.cif

Structure factors: contains datablocks I. DOI: 10.1107/S1600536808044267/hk2578Isup2.hkl

Additional supplementary materials:  crystallographic information; 3D view; checkCIF report

## Figures and Tables

**Table 1:** Selected geometric parameters (Å, °)

Ca1—O4^i^	2.366 (3)
Ca1—O5	2.373 (3)
Ca1—O1	2.375 (2)
Ca1—O3	2.391 (3)
Ca1—O6	2.393 (3)
Ca1—O2^ii^	2.461 (3)
Ca1—O1^ii^	2.641 (3)
Ca1—O4	2.734 (3)

Symmetry codes: (i) 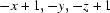; (ii) 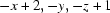.

**Table 2:** Hydrogen-bond geometry (Å, °)

*D*—H⋯*A*	*D*—H	H⋯*A*	*D*⋯*A*	*D*—H⋯*A*
O5—H5*B*⋯O3^ii^	0.85	1.92	2.765 (3)	171
O5—H5*C*⋯N2^iii^	0.85	2.01	2.831 (3)	162
O6—H6*B*⋯N1^iv^	0.85	2.07	2.919 (4)	173
O6—H6*C*⋯O2^v^	0.85	2.00	2.826 (3)	165
C6—H6⋯O5^iv^	0.93	2.52	3.432 (4)	166

Symmetry codes: (ii) 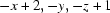; (iii) 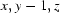; (iv) 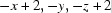; (v) 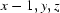.

**Fig. 1.**
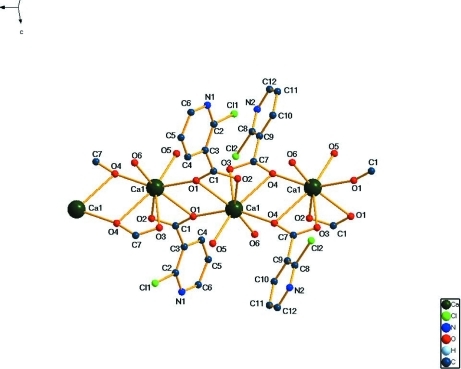
The molecular structure of the title molecule, with the atom-numbering scheme. Displacement ellipsoids are drawn at the 30% probability level.

**Fig. 2.**
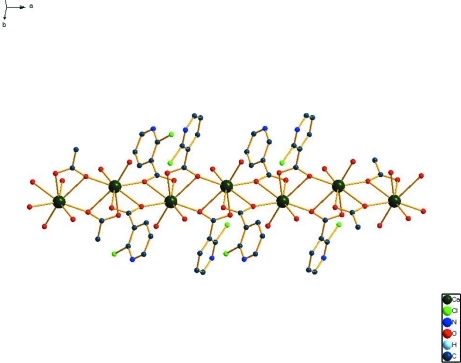
A partial packing diagram of the title compound, showing the formation of the one dimensional chain structure.

## supplementary crystallographic information

### Comment

Chemistry of alkaline earth metals is an unexplored area. The model complexes containing Mg^2+^ and Ca^2+^ cations have previously been prepared and used as probes for understanding the binding modes of these metals (Schmidbaur *et al.*,  1989, 1990). In our ongoing studies with 2-chloronicotinate ligand and s-block metal ions, the title compound has been synthesized, and we report herein its crystal structure.

In the molecule of the title compound, (I), (Fig. 1), the bond lengths (Allen *et al.*,  1987) and angles are within normal ranges. It has an inversion centre midway between the two Ca^II^ ions, which are bridged by 2-chloronicotinate groups. Each Ca atom is eight-coordinated by six O atoms of 2-chloronicotinate ligands and two O atoms of water molecules. It essentially forms a one-dimensional chain structure (Fig. 2). The Ca—O bonds are in the range of [2.366 (3)–2.734 (3) Å] (Table 1). The average value of the Ca—O bonds [2.467 (3) Å] is almost the same with the corresponding values [2.4674 (9) Å] in [Ca(3-aba)_2_(H_2_O)_2_]_n_ (where 3-aba is 3-aminobenzoic acid), (II) (Murugavel & Banerjee, 2003) and [2.4556 (8) Å] in [Ca(H_2_O)_3_Ca(1,3-pdta)- (H_2_O)]. 2(H_2_O) (where 1,3-pdta is the 1,3-propanediaminetetraacetate ion), (III) (Radanovic *et al.*,  2004). The Ca1—Ca1^i^ [4.0553 (14) Å] and Ca1—Ca1^ii^ [4.0681 (14) Å] [symmetry codes: (i) 1 - x, -y, 1 - z, (ii) 2 - x, -y, 1 - z] distances are longer than the corresponding value [4.0034 (5) Å] in (II).

In the crystal structure, intermolecular O—H···O, O—H···N and C—H···O hydrogen bonds (Table 2) result in the formation of a supramolecular network structure. The π–π contacts between the 2-chloronicotinate rings, Cg1–Cg1^i^ and Cg2–Cg2^ii^ [symmetry codes: (i) -x, 2 - y, -z; (ii) 1 - x, 1 - y, 1 - z, where Cg1 and Cg2 are centroids of the rings A (N1/C2–C6) and B (N2/C8–C12), respectively] may further stabilize the structure, with centroid–centroid distances of 3.875 (3) Å and 3.701 (3) Å.

### Experimental

For the preparation of the title compound, CaCl_2_(H_2_O)_2_ (0.588 g, 4 mmol) and 2-chloronicotinic acid (0.044 g, 0.4 mmol) were dissolved in H_2_O (20 ml) and MeOH (30 ml) by refluxing for 30 min. Sodium methoxide (0.4 mmol, 0.8 ml) was added dropwise by stirring. After refluxing for 8 h, the colorless solution was obtained, and then filtered. The solvent was gradually removed by evaporation under vacuum until the colorless solid was obtained, which was recrystallized from petroleum ether–dichoromethane (1:1) to give the block-shaped colorless crystals.

### Refinement

H atoms were positioned geometrically, with O—H = 0.85 Å (for H_2_O) and C—H = 0.93 Å for aromatic H, respectively, and constrained to ride on their parent atoms with U_iso_(H) = 1.2U_eq_(C,O).

### Crystal data

**Table:**

[Ca(C_6_H_3_ClNO_2_)_2_(H_2_O)_2_]	*Z* = 2
*M**_r_* = 389.20	*F*(000) = 396
Triclinic, *P*1	*D*_x_ = 1.442 Mg m^−^^3^
Hall symbol: -P 1	Mo *K*α radiation, λ = 0.71073 Å
*a* = 7.8363 (10) Å	Cell parameters from 2126 reflections
*b* = 10.8421 (16) Å	θ = 2.5–27.6°
*c* = 10.8834 (16) Å	µ = 0.68 mm^−^^1^
α = 98.455 (2)°	*T* = 298 K
β = 97.610 (1)°	Block, colourless
γ = 97.289 (1)°	0.48 × 0.40 × 0.30 mm
*V* = 896.4 (2) Å^3^	

### Data collection

**Table:**

Bruker SMART CCD area-detector diffractometer	3074 independent reflections
Radiation source: fine-focus sealed tube	2306 reflections with *I* > 2σ(*I*)
graphite	*R*_int_ = 0.022
φ and ω scans	θ_max_ = 25.0°, θ_min_ = 1.9°
Absorption correction: multi-scan (*SADABS*; Sheldrick, 1996)	*h* = −9→9
*T*_min_ = 0.738, *T*_max_ = 0.823	*k* = −11→12
4594 measured reflections	*l* = −11→12

### Refinement

**Table:**

Refinement on *F*^2^	Primary atom site location: structure-invariant direct methods
Least-squares matrix: full	Secondary atom site location: difference Fourier map
*R*[*F*^2^ > 2σ(*F*^2^)] = 0.043	Hydrogen site location: inferred from neighbouring sites
*wR*(*F*^2^) = 0.145	H-atom parameters constrained
*S* = 1.03	*w* = 1/[σ^2^(*F*_o_^2^) + (0.072*P*)^2^ + 0.8771*P*] where *P* = (*F*_o_^2^ + 2*F*_c_^2^)/3
3074 reflections	(Δ/σ)_max_ = 0.001
208 parameters	Δρ_max_ = 0.47 e Å^−^^3^
0 restraints	Δρ_min_ = −0.45 e Å^−^^3^

### Special details

**Table:**

Geometry. All esds (except the esd in the dihedral angle between two l.s. planes) are estimated using the full covariance matrix. The cell esds are taken into account individually in the estimation of esds in distances, angles and torsion angles; correlations between esds in cell parameters are only used when they are defined by crystal symmetry. An approximate (isotropic) treatment of cell esds is used for estimating esds involving l.s. planes.
Refinement. Refinement of F^2^ against ALL reflections. The weighted R-factor wR and goodness of fit S are based on F^2^, conventional R-factors R are based on F, with F set to zero for negative F^2^. The threshold expression of F^2^ > 2sigma(F^2^) is used only for calculating R-factors(gt) etc. and is not relevant to the choice of reflections for refinement. R-factors based on F^2^ are statistically about twice as large as those based on F, and R- factors based on ALL data will be even larger.

### Fractional atomic coordinates and isotropic or equivalent isotropic displacement parameters (Å^2^)

**Table:**

	*x*	*y*	*z*	*U*_iso_*/*U*_eq_	
Ca1	0.75350 (8)	−0.02117 (7)	0.54151 (6)	0.0273 (2)	
Cl1	1.31442 (17)	−0.08155 (14)	0.89399 (12)	0.0688 (4)	
Cl2	0.85580 (16)	0.45842 (12)	0.62037 (12)	0.0642 (4)	
N1	1.2104 (5)	0.0770 (5)	1.0606 (3)	0.0597 (11)	
N2	0.7024 (5)	0.5552 (3)	0.4414 (4)	0.0521 (9)	
O1	1.0474 (3)	0.0510 (2)	0.6352 (2)	0.0363 (6)	
O2	1.3309 (3)	0.1026 (3)	0.6846 (2)	0.0468 (7)	
O3	0.8087 (3)	0.1815 (3)	0.4807 (3)	0.0501 (8)	
O4	0.5271 (3)	0.1368 (3)	0.4706 (3)	0.0447 (7)	
O5	0.8604 (3)	−0.2084 (2)	0.5835 (2)	0.0395 (6)	
H5B	0.9605	−0.2092	0.5617	0.047*	
H5C	0.7926	−0.2722	0.5410	0.047*	
O6	0.6803 (4)	0.0628 (3)	0.7404 (3)	0.0539 (8)	
H6B	0.7060	0.0164	0.7946	0.065*	
H6C	0.5721	0.0672	0.7340	0.065*	
C1	1.1815 (5)	0.0877 (4)	0.7134 (3)	0.0336 (8)	
C2	1.2213 (5)	0.0517 (4)	0.9394 (4)	0.0447 (10)	
C3	1.1612 (5)	0.1197 (4)	0.8500 (3)	0.0392 (9)	
C4	1.0797 (6)	0.2201 (5)	0.8907 (4)	0.0574 (12)	
H4	1.0330	0.2677	0.8339	0.069*	
C5	1.0684 (7)	0.2488 (6)	1.0167 (5)	0.0694 (15)	
H5	1.0168	0.3176	1.0462	0.083*	
C6	1.1333 (6)	0.1758 (6)	1.0973 (4)	0.0644 (14)	
H6	1.1235	0.1957	1.1819	0.077*	
C7	0.6592 (5)	0.2077 (3)	0.4592 (4)	0.0335 (8)	
C8	0.7209 (5)	0.4468 (4)	0.4786 (4)	0.0404 (9)	
C9	0.6374 (4)	0.3299 (3)	0.4140 (4)	0.0334 (8)	
C10	0.5307 (5)	0.3306 (4)	0.3029 (4)	0.0491 (11)	
H10	0.4696	0.2553	0.2566	0.059*	
C11	0.5145 (6)	0.4422 (5)	0.2605 (5)	0.0595 (13)	
H11	0.4454	0.4430	0.1844	0.071*	
C12	0.6014 (6)	0.5528 (5)	0.3319 (5)	0.0598 (13)	
H12	0.5896	0.6283	0.3031	0.072*	

### Atomic displacement parameters (Å^2^)

**Table:**

	*U*^11^	*U*^22^	*U*^33^	*U*^12^	*U*^13^	*U*^23^
Ca1	0.0220 (4)	0.0305 (4)	0.0307 (4)	0.0046 (3)	0.0048 (3)	0.0083 (3)
Cl1	0.0708 (8)	0.0885 (10)	0.0633 (8)	0.0386 (7)	0.0216 (6)	0.0320 (7)
Cl2	0.0589 (7)	0.0549 (7)	0.0694 (8)	0.0096 (6)	−0.0128 (6)	−0.0008 (6)
N1	0.040 (2)	0.105 (3)	0.033 (2)	0.008 (2)	0.0044 (16)	0.013 (2)
N2	0.042 (2)	0.0327 (19)	0.083 (3)	0.0083 (16)	0.0124 (19)	0.0121 (18)
O1	0.0253 (13)	0.0476 (16)	0.0334 (14)	0.0041 (11)	0.0003 (11)	0.0036 (12)
O2	0.0246 (14)	0.077 (2)	0.0374 (15)	0.0034 (13)	0.0070 (11)	0.0061 (14)
O3	0.0299 (15)	0.0408 (16)	0.088 (2)	0.0111 (12)	0.0138 (14)	0.0274 (15)
O4	0.0327 (15)	0.0402 (15)	0.0632 (19)	−0.0010 (12)	0.0142 (13)	0.0149 (13)
O5	0.0300 (14)	0.0340 (14)	0.0570 (17)	0.0060 (11)	0.0099 (12)	0.0119 (12)
O6	0.0371 (16)	0.089 (2)	0.0397 (16)	0.0195 (15)	0.0091 (13)	0.0113 (15)
C1	0.031 (2)	0.041 (2)	0.0295 (19)	0.0066 (16)	0.0028 (16)	0.0084 (16)
C2	0.031 (2)	0.070 (3)	0.034 (2)	0.0077 (19)	0.0065 (16)	0.009 (2)
C3	0.0250 (19)	0.057 (3)	0.033 (2)	0.0035 (17)	0.0048 (16)	0.0027 (18)
C4	0.060 (3)	0.068 (3)	0.045 (3)	0.017 (2)	0.011 (2)	0.005 (2)
C5	0.064 (3)	0.088 (4)	0.053 (3)	0.019 (3)	0.018 (3)	−0.012 (3)
C6	0.048 (3)	0.104 (4)	0.036 (3)	0.004 (3)	0.012 (2)	−0.005 (3)
C7	0.0277 (19)	0.0288 (19)	0.045 (2)	0.0062 (15)	0.0093 (16)	0.0057 (16)
C8	0.030 (2)	0.034 (2)	0.059 (3)	0.0058 (16)	0.0092 (18)	0.0109 (18)
C9	0.0230 (18)	0.033 (2)	0.047 (2)	0.0064 (15)	0.0110 (16)	0.0109 (17)
C10	0.043 (2)	0.044 (2)	0.060 (3)	0.0105 (19)	0.001 (2)	0.009 (2)
C11	0.067 (3)	0.057 (3)	0.056 (3)	0.019 (2)	−0.005 (2)	0.020 (2)
C12	0.056 (3)	0.048 (3)	0.082 (4)	0.014 (2)	0.010 (3)	0.029 (3)

### Geometric parameters (Å, °)

**Table:**

Ca1—O4^i^	2.366 (3)	O4—C7	1.241 (4)
Ca1—O5	2.373 (3)	O4—Ca1^i^	2.366 (3)
Ca1—O1	2.375 (2)	O5—H5B	0.8499
Ca1—O3	2.391 (3)	O5—H5C	0.8500
Ca1—O6	2.393 (3)	O6—H6B	0.8501
Ca1—O2^ii^	2.461 (3)	O6—H6C	0.8500
Ca1—O1^ii^	2.641 (3)	C1—C3	1.511 (5)
Ca1—O4	2.734 (3)	C1—Ca1^ii^	2.890 (4)
Ca1—C1^ii^	2.890 (4)	C2—C3	1.375 (6)
Ca1—Ca1^i^	4.0553 (14)	C3—C4	1.377 (6)
Ca1—Ca1^ii^	4.0681 (14)	C4—C5	1.378 (6)
Ca1—H5B	2.7769	C4—H4	0.9300
Ca1—H5C	2.7764	C5—C6	1.357 (8)
Cl1—C2	1.737 (5)	C5—H5	0.9300
Cl2—C8	1.729 (4)	C6—H6	0.9300
N1—C2	1.323 (5)	C7—C9	1.501 (5)
N1—C6	1.332 (7)	C8—C9	1.391 (5)
N2—C8	1.317 (5)	C9—C10	1.378 (5)
N2—C12	1.336 (6)	C10—C11	1.372 (6)
O1—C1	1.242 (4)	C10—H10	0.9300
O1—Ca1^ii^	2.641 (3)	C11—C12	1.372 (7)
O2—C1	1.248 (4)	C11—H11	0.9300
O2—Ca1^ii^	2.461 (3)	C12—H12	0.9300
O3—C7	1.241 (4)		
			
O4^i^—Ca1—O5	85.90 (9)	O5—Ca1—H5C	16.8
O4^i^—Ca1—O1	154.07 (10)	O1—Ca1—H5C	92.6
O5—Ca1—O1	76.49 (9)	O3—Ca1—H5C	155.0
O4^i^—Ca1—O3	124.46 (9)	O6—Ca1—H5C	108.6
O5—Ca1—O3	148.15 (9)	O2^ii^—Ca1—H5C	80.4
O1—Ca1—O3	77.33 (9)	O1^ii^—Ca1—H5C	80.4
O4^i^—Ca1—O6	79.48 (10)	O4—Ca1—H5C	144.2
O5—Ca1—O6	102.23 (10)	C1^ii^—Ca1—H5C	80.4
O1—Ca1—O6	85.76 (9)	Ca1^i^—Ca1—H5C	111.8
O3—Ca1—O6	93.57 (11)	Ca1^ii^—Ca1—H5C	85.3
O4^i^—Ca1—O2^ii^	76.74 (9)	H5B—Ca1—H5C	28.7
O5—Ca1—O2^ii^	93.19 (10)	C2—N1—C6	116.7 (4)
O1—Ca1—O2^ii^	122.53 (9)	C8—N2—C12	117.9 (4)
O3—Ca1—O2^ii^	85.84 (11)	C1—O1—Ca1	162.8 (2)
O6—Ca1—O2^ii^	150.55 (10)	C1—O1—Ca1^ii^	88.6 (2)
O4^i^—Ca1—O1^ii^	124.14 (9)	Ca1—O1—Ca1^ii^	108.26 (9)
O5—Ca1—O1^ii^	80.03 (9)	C1—O2—Ca1^ii^	96.9 (2)
O1—Ca1—O1^ii^	71.74 (9)	C7—O3—Ca1	101.6 (2)
O3—Ca1—O1^ii^	74.78 (9)	C7—O4—Ca1^i^	168.0 (3)
O6—Ca1—O1^ii^	156.33 (9)	C7—O4—Ca1	85.3 (2)
O2^ii^—Ca1—O1^ii^	50.80 (8)	Ca1^i^—O4—Ca1	105.13 (10)
O4^i^—Ca1—O4	74.87 (10)	Ca1—O5—H5B	109.6
O5—Ca1—O4	160.24 (9)	Ca1—O5—H5C	109.6
O1—Ca1—O4	123.16 (9)	H5B—O5—H5C	108.3
O3—Ca1—O4	49.89 (8)	Ca1—O6—H6B	110.5
O6—Ca1—O4	79.04 (9)	Ca1—O6—H6C	110.5
O2^ii^—Ca1—O4	78.19 (10)	H6B—O6—H6C	108.8
O1^ii^—Ca1—O4	106.86 (8)	O1—C1—O2	123.5 (3)
O4^i^—Ca1—C1^ii^	100.91 (10)	O1—C1—C3	117.9 (3)
O5—Ca1—C1^ii^	87.26 (10)	O2—C1—C3	118.5 (3)
O1—Ca1—C1^ii^	97.14 (10)	O1—C1—Ca1^ii^	65.98 (19)
O3—Ca1—C1^ii^	78.32 (11)	O2—C1—Ca1^ii^	57.69 (19)
O6—Ca1—C1^ii^	170.49 (11)	C3—C1—Ca1^ii^	175.5 (3)
O2^ii^—Ca1—C1^ii^	25.39 (9)	N1—C2—C3	125.2 (4)
O1^ii^—Ca1—C1^ii^	25.45 (9)	N1—C2—Cl1	115.4 (3)
O4—Ca1—C1^ii^	91.86 (9)	C3—C2—Cl1	119.4 (3)
O4^i^—Ca1—Ca1^i^	40.60 (7)	C2—C3—C4	116.7 (4)
O5—Ca1—Ca1^i^	126.35 (7)	C2—C3—C1	122.6 (4)
O1—Ca1—Ca1^i^	153.30 (7)	C4—C3—C1	120.7 (4)
O3—Ca1—Ca1^i^	84.02 (7)	C3—C4—C5	119.0 (5)
O6—Ca1—Ca1^i^	76.43 (7)	C3—C4—H4	120.5
O2^ii^—Ca1—Ca1^i^	74.23 (6)	C5—C4—H4	120.5
O1^ii^—Ca1—Ca1^i^	121.53 (6)	C6—C5—C4	119.5 (5)
O4—Ca1—Ca1^i^	34.28 (5)	C6—C5—H5	120.3
C1^ii^—Ca1—Ca1^i^	97.60 (7)	C4—C5—H5	120.3
O4^i^—Ca1—Ca1^ii^	152.81 (8)	N1—C6—C5	122.9 (4)
O5—Ca1—Ca1^ii^	75.59 (6)	N1—C6—H6	118.5
O1—Ca1—Ca1^ii^	38.06 (6)	C5—C6—H6	118.5
O3—Ca1—Ca1^ii^	72.63 (7)	O3—C7—O4	123.2 (3)
O6—Ca1—Ca1^ii^	123.46 (8)	O3—C7—C9	118.2 (3)
O2^ii^—Ca1—Ca1^ii^	84.47 (6)	O4—C7—C9	118.6 (3)
O1^ii^—Ca1—Ca1^ii^	33.68 (5)	N2—C8—C9	124.5 (4)
O4—Ca1—Ca1^ii^	120.51 (6)	N2—C8—Cl2	114.9 (3)
C1^ii^—Ca1—Ca1^ii^	59.09 (7)	C9—C8—Cl2	120.6 (3)
Ca1^i^—Ca1—Ca1^ii^	149.45 (4)	C10—C9—C8	116.3 (3)
O4^i^—Ca1—H5B	101.0	C10—C9—C7	120.2 (3)
O5—Ca1—H5B	16.8	C8—C9—C7	123.5 (3)
O1—Ca1—H5B	64.9	C11—C10—C9	120.0 (4)
O3—Ca1—H5B	131.5	C11—C10—H10	120.0
O6—Ca1—H5B	111.9	C9—C10—H10	120.0
O2^ii^—Ca1—H5B	89.7	C10—C11—C12	119.2 (4)
O1^ii^—Ca1—H5B	65.5	C10—C11—H11	120.4
O4—Ca1—H5B	167.8	C12—C11—H11	120.4
C1^ii^—Ca1—H5B	77.5	N2—C12—C11	122.0 (4)
Ca1^i^—Ca1—H5B	140.4	N2—C12—H12	119.0
Ca1^ii^—Ca1—H5B	58.9	C11—C12—H12	119.0
O4^i^—Ca1—H5C	72.5		

Symmetry codes: (i) −*x*+1, −*y*, −*z*+1; (ii) −*x*+2, −*y*, −*z*+1.

### Hydrogen-bond geometry (Å, °)

**Table:**

*D*—H···*A*	*D*—H	H···*A*	*D*···*A*	*D*—H···*A*
O5—H5B···O3^ii^	0.85	1.92	2.765 (3)	171
O5—H5C···N2^iii^	0.85	2.01	2.831 (3)	162
O6—H6B···N1^iv^	0.85	2.07	2.919 (4)	173
O6—H6C···O2^v^	0.85	2.00	2.826 (3)	165
C6—H6···O5^iv^	0.93	2.52	3.432 (4)	166

Symmetry codes: (ii) −*x*+2, −*y*, −*z*+1; (iii) *x*, *y*−1, *z*; (iv) −*x*+2, −*y*, −*z*+2; (v) *x*−1, *y*, *z*.
